# Meta-transcriptomic profiling of *Ixodes ricinus and Ixodes persulcatus* ticks from Finland reveals diverse RNA viromes, community structuring, and emerging viral lineages

**DOI:** 10.1007/s00705-026-06741-y

**Published:** 2026-09-21

**Authors:** Theophilus Yaw Alale, Jesse Mänttäri, Heidi M. Viitaniemi, Mikhail Ozerov, Miguel Baltazar-Soares, Jani J. Sormunen, Eero J. Vesterinen

**Affiliations:** 1 Department of Biology, University of Turku, Turku, Finland; 2 Bioname Ltd, Itäinen Pitkäkatu 2, Turku, 20520 Finland; 3 School of Medical, Molecular and Forensic Sciences, Murdoch University, Perth, WA Australia

## Abstract

Tick-borne viruses (TBVs) threaten human and animal health in northern Europe, where *Ixodes ricinus* and *Ixodes persulcatus* are expanding their range. In Finland, the genetic diversity, population structure, and tick-associated RNA virome of these species remain insufficiently characterized. Characterizing tick-borne viruses in Finland is essential to detect emerging pathogens at the northern edge of tick expansion, where climate-driven ecological change may facilitate the spread of novel viruses with potential risks to human and animal health. We applied single-tick metatranscriptomic approach to profile and compare the RNA viromes of 92 adult *I. ricinus* and *I. persulcatus* collected from two locations in Finland. We identified 42 RNA viruses spanning 12 families, with *Nairoviridae*,* Phenuiviridae*, *Partitiviridae*, *Flaviviridae*, and *Rhabdoviridae* being the most represented. Viral community composition differed significantly between tick species, with *I. persulcatus* exhibiting more constrained but *bunyaviridae*-dominated viromes, whereas *I. ricinus* showed greater inter-individual variability and enrichment of partiti-like and iflavirus taxa. Phylogenetic reconstruction of conserved viral proteins supported the presence of several divergent viral lineages and confirmed the first detection of multiple Eurasian tick-associated viruses in Finland, including Nuomin virus, Beiji nairovirus, and Onega tick phlebovirus. Several detected viruses clustered within clades that include known or suspected zoonotic agents, highlighting the need for close monitoring of tick populations, especially in zones of sympatric occurrence where hybridization and associated close contact between the tick species have been observed. These findings reveal substantial, species-structured viral diversity in Finnish *Ixodes* ticks.

**Supplementary Information:**

The online version contains supplementary material available at https://doi.org/10.1007/s00705-026-06741-y.

## Introduction

Ticks are obligate hematophagous ectoparasites of a wide range of vertebrate hosts, including mammals, birds, and reptiles. They are considered the second most important vectors of human and animal pathogens worldwide after mosquitoes [9], [35], [56]. More than 900 tick species have been described, most belonging to the *Ixodidae* (hard ticks) and *Argasidae* (soft ticks) families [24]. Many of these species are of significant veterinary and public health importance due to their role in transmitting a diverse range of pathogens, including bacteria (e.g., *Borrelia*, *Anaplasma*), protozoa (e.g., *Babesia*), and numerous viruses (e.g., tick-borne encephalitis virus).

In northern Europe, *Ixodes ricinus* (the castor bean tick) and *I. persulcatus* (the taiga tick) are the dominant tick species associated with the transmission of tick-borne pathogens [36], [42]. *Ixodes ricinus* is widespread across Western and Central Europe, extending into parts of northern Europe, including southern and coastal Finland, whereas *I. persulcatus* is distributed across northern and eastern Europe and in Asia [15, 18]. These species overlap geographically in parts of Finland, Estonia and Russia, forming hybrid zones that may create opportunities for cross-species tick-borne pathogen exchange and genetic introgression among tick populations [1]. Both species exhibit broad host ranges, infesting humans, livestock, companion animals, and wildlife [28], [51] making them central to the ecology of many vector-borne pathogens. Their overlapping habitats in Finland, combined with changing environmental conditions, have been linked to the increased prevalence and northward spread of tick-borne pathogens [25], [71]. Tick-borne viruses (TBVs) represent a rapidly expanding group of emerging infectious agents, several of which can cause severe diseases in humans and domestic animals. These viruses include members of several RNA virus families, such as Flaviviridae, *Nairoviridae*, *Phenuiviridae*, *Rhabdoviridae*, *Reoviridae*, and *Orthomyxoviridae* [6], [13], [55], [57]. Notable TBVs of medical and veterinary importance in Europe include TBEV, Louping ill virus and Crimean-Congo hemorrhagic fever virus (CCHFV) [13], [18]. In Finland, the most significant TBVs reported to date include TBEV [25] and the recently identified Alongshan virus (ALSV) [34]. These viruses can cause a spectrum of diseases ranging from mild febrile illness to severe neurological disorders and haemorrhagic fevers, underscoring their public health importance. Given Finland’s northern latitude, extensive forest coverage, and seasonal temperature variation, the region provides a unique ecological setting to study the diversity and evolution of TBVs under the influence of climatic and ecological change [62], [71]. The increasing discovery of novel tick-associated viruses across Eurasia underscores the need for continued surveillance of viral diversity in regions undergoing ecological and climatic transition [56], 72]. Therefore, understanding the composition of the virome of *I. ricinus* and *I. persulcatus* in Finland is particularly important due to the recent expansion in distributions, where climate change is altering tick abundance, host availability, and species overlap [2, 19, 58]. Additionally, sympatric zones may promote viral exchange, reshape viral community structure and potentially influence transmission dynamics [14, 61]. Without such regional baselines, it may be difficult to detect shifts in viral diversity associated with climate change, range expansion, or ecological disturbance.

Single-tick metatranscriptomics enables a fine-scale understanding of the tick virome, diversity and structure, revealing how viral communities differ between species, sexes, and environments [45]. The application of metatranscriptomics to individual ticks has proven particularly powerful for resolving inter-individual variation in viral carriage, co-infections, and abundance patterns [52], [66]. Understanding these factors is essential for predicting viral emergence and informing effective surveillance and control measures. Although metatranscriptomic studies of tick-borne viruses have been conducted elsewhere in Europe, they have focused primarily on *I. ricinus* [52], leaving comparative data for *I. persulcatus* limited. Comprehensive virome profiling across distinct ecological zones is therefore critical to assess species-specific viral structure, identify potential zoonotic threats, and clarify evolutionary relationships among tick-associated viruses.

In this study, we applied a single-tick metatranscriptomic (RNA-based metaviromic) approach to investigate the viral communities of *I. ricinus* and *I. persulcatus* collected from two geographically distinct Finnish populations in Ruissalo (Turku) and Hervanta (Tampere). Specifically, we aimed to (1) identify and phylogenetically classify tick-associated RNA viruses; (2) comprehensively characterize and compare the RNA viromes of the two tick species; and (3) quantify species-specific differences in alpha and beta diversity, viral abundance, and community structure.

## Materials and methods

### Sample collection and RNA extraction

Adult ticks of both species were collected by cloth dragging from two geographically distinct populations: *I. ricinus* from Ruissalo Island near the city of Turku (60.42211° N, 22.09654° E) and *I. persulcatus* from Finland’s southernmost known population in Hervanta, Tampere (61.43328° N, 23.83003° E). Collections were done in May-July 2024. Ticks were collected into falcon tubes and kept alive in a chamber at room temperature with > 85% relative humidity (RH) to prevent desiccation. A total of 96 individual ticks (44 males and 52 females) were processed for metatranscriptomic analysis. Prior to RNA extraction, each tick was washed sequentially with 70% ethanol and rinsed with nuclease-free water to remove surface contaminants. Individual ticks were then flash-frozen in liquid nitrogen and homogenized in lysis buffer using sterile plastic pestles following the manufacturer’s protocol for the Zymo Research Quick-RNA™ MiniPrep Kit (catalogue number: D7001). Total RNA was extracted from each tick, eluted in 50 µL of nuclease-free water, and stored at -80 °C until library preparation. RNA purity and yield were measured using both NanoDrop spectrophotometer and Qubit 4 Fluorometer (Thermo Fisher Scientific).

### Ribosomal RNA depletion

To enrich for viral and non-ribosomal transcripts, ribosomal RNA was depleted using the NEBNext^®^ rRNA Depletion Kit v2 (Human/Mouse/Rat) (New England Biolabs, #E7400). The depletion protocol was carried out according to the manufacturer’s guidelines, effectively removing cytoplasmic and mitochondrial rRNA. The rRNA treatment protocol also included RNAse and DNAse treatment steps. The resulting rRNA-depleted RNA was purified using Agencourt RNAClean XP beads (Beckman Coulter) and quantified with a Qubit RNA HS Assay Kit (Thermo Fisher Scientific).

### Library preparation and sequencing

Metatranscriptomic libraries were prepared from rRNA-depleted RNA using the NEBNext^®^ Ultra II Directional RNA Library Prep Kit (New England Biolabs, #E7760L). RNA fragmentation, first- and second-strand cDNA synthesis, adapter ligation, and PCR enrichment were performed according to the manufacturer’s recommendations, with PCR cycle numbers optimized (12 cycles for 100 ng of total RNA) to minimize amplification bias. Library size distribution was confirmed on an Agilent 2100 Bioanalyzer, and concentrations were measured using a Qubit dsDNA HS Assay Kit. Fragment size selection was performed using E-Gel SizeSelect™ II (Invitrogen) following the manufacturer’s recommendations to obtain insert sizes of 300–450 bp.

Libraries were pooled equimolarly and sequenced on an Illumina NovaSeq 6000 platform at the Finnish Functional Genomics Centre, University of Turku, generating paired-end 150 bp reads. Sequencing depth was optimized to capture low-abundance viral transcripts.

### Bioinformatics workflow

All computational analyses were conducted on the CSC Puhti supercomputing cluster (CSC - IT Center for Science, Finland). Data processing, assembly, annotation, and statistical analyses were performed using a combination of custom bash scripts, R scripts, and standardized bioinformatics workflows.

#### Quality control and read filtering

Raw reads were quality-checked using FastQC (v0.11.9) [5] and trimmed with Trimmomatic (v0.39, Java 11) [7] to remove adapters and low-quality bases (SLIDINGWINDOW:4:20, MINLEN:50). Only paired reads were used. Trimmed read quality was reassessed using MultiQC [21].

#### Tick host read removal

Trimmed reads were mapped to species-specific reference genomes of *I. ricinus* (GenBank accession numbers PRJNA270959) [16] and *I. persulcatus* (GenBank accession number PRJNA633311 [27] using HISAT2 (--rna-strandness FR) (v2.2.1) [32]. Concordantly mapped reads were removed, and unmapped paired-end reads were retained for downstream metatranscriptomic analysis. Mapping statistics were extracted from alignment summary files and aggregated across samples.

#### Taxonomic classification

Unmapped reads were taxonomically classified using Kraken2 (v2.1.3) [70] which uses a custom k-mer-based NCBI RefSeq database containing viral, bacterial, protozoan, and archaeal genomes (updated April 2024). Classification confidence was set at 0.1. Read counts were refined using Bracken (v2.8) to estimate accurate species-level abundance. Classification reports were processed using Bracken to estimate taxon-level relative abundances at species and genus levels. Kraken2 outputs were parsed using custom Python scripts to generate per-sample taxonomic tables, which were merged into a unified abundance matrix for downstream statistical analyses.

#### Extraction of viral reads

Virus-assigned reads were extracted following the National Microbiome Data Collaborative (NMDC) (https://nmdc-edge.org) [17], [30] metagenomic workflow framework. Reads identified with Kraken2 classification outputs were extracted using KrakenTools (extract_kraken_reads.py). Those reads assigned to NCBI TaxID 10,239 (Viruses) and to viral families of public health interest (including *Nairoviridae*,* Phenuiviridae*, *Flaviviridae*, *Partitiviridae*, *Chuviridae*) were extracted into family-specific paired FASTQ files using automated Bash workflows. This hierarchical extraction strategy improves sensitivity for low-abundance viral taxa and reduces assembly complexity in mixed-community datasets [54].

#### De novo viral assembly

Extracted viral reads were assembled using rnaSPAdes (v3.15.5) [11] under the default parameter for stranded data. Assemblies were conducted both at the individual-sample level and, where appropriate, using pooled family-level viral read sets to enhance recovery of fragmented viral genomes. Only individual-level sample assembly data were used for downstream analysis. Contigs shorter than 1000 nucleotides were excluded from further analysis to reduce spurious assemblies and non-informative fragments.

Assembled viral contigs were evaluated using CheckV [48] and QUAST v5.2.0 [23], which provided assembly statistics such as N50, contig length distribution, and genome completeness estimates, contamination, and viral host integration signals. Viral contigs were categorized into quality tiers (complete, high-quality, medium-quality, or low-quality) following NMDC viral genome reporting standards. Only contigs meeting required minimum completeness and quality thresholds (complete:100% completeness, high-quality: ≥90% completeness, medium-quality: 50–90% completeness, low-quality: <50% completeness) were retained for taxonomic classification and phylogenetic inference [41].

#### Viral gene prediction and functional annotation

Genome annotation and functional characterization of viral contigs were performed using the standardized NMDC metagenomic annotation pipeline (https://nmdc-edge.org/home) [17], [30]. Open reading frames (ORFs) were predicted using Prodigal in metagenomic mode. Predicted proteins were annotated using BLASTp [3] searches against the NCBI RefSeq viral protein database, HMMER [22] searches against Pfam and viral protein family HMM profiles, and eggNOG-mapper (v2) [12] for functional classification and ortholog assignment.

Taxonomic assignment of viral contigs was refined using combined evidence from BLAST using a minimum of 70% identity and less than 1 × 10^− 5^ similarity threshold, conserved domain architecture, and phylogenetic placement. Viral family classification followed the International Committee on Taxonomy of Viruses (ICTV) [38] consistent taxonomy used within the NMDC framework.

#### Taxonomic confirmation and novel virus assessment

Taxonomic assignments were confirmed using reciprocal best-hit BLAST strategies and phylogenetic placement of conserved proteins, particularly RNA-dependent RNA polymerase (RdRp). Viral sequences showing < 75% amino acid identity to known references and forming monophyletic clades distinct from recognized species were classified as divergent or putative novel viral lineages, consistent with ICTV sequence demarcation guidelines and NMDC viral genome reporting criteria. Final viral classifications were cross-validated against GenBank and recent tick virome datasets.

### Statistical analyses and visualization

All statistical analyses and plotting were conducted in R (v4.3.2) [53] using a combination of base functions and Bioconductor packages. Viral abundance matrices generated from the Kraken2 and Bracken outputs were imported into R and normalized before analysis. Data manipulation and summarization were performed using the tidyverse package [69]. To assess within-sample diversity, alpha diversity indices including Shannon, Simpson, and species richness were calculated using the vegan R package (v2.8-0) [3]. These indices provided insights into viral community complexity and evenness within each tick sample.

#### Viral community and diversity analyses

Viral abundance tables derived from Kraken2 output and viral families based on taxonomic classification were merged into a unified abundance matrix. Alpha-diversity metrics (Shannon and Simpson indices) were calculated using the vegan R package [26]. Beta-diversity was assessed using Bray-Curtis dissimilarity, and differences in community composition were tested using PERMANOVA with 999 permutations [4]. Non-metric multidimensional scaling (NMDS) ordination was used to visualize inter-sample relationships. Heatmaps of viral relative abundance were generated using hierarchical clustering based on Bray-Curtis distances.

#### Differential abundance analysis

Differential viral abundance between tick species was assessed using DESeq2 (v1.42.0) [43] on raw count matrices derived from classified viral reads. Counts with at least 1000 viral reads were normalized using the median-of-ratios method. Viruses with adjusted p-values (Benjamini-Hochberg) below 0.05 were considered significantly differentially abundant. Log2 fold changes were used to quantify the magnitude and direction of species-associated differences in viral abundance.

### Phylogenetic analysis

To infer the evolutionary relationships of viral sequences detected in tick samples, phylogenetic analyses were conducted using RNA-dependent RNA polymerase (RdRp) amino acid sequences, which represent the most conserved genomic region across RNA viruses and are widely used for virus classification. Viral contigs containing putative RdRp domains were identified from de novo assemblies based on DIAMOND BLASTp similarity searches against the NCBI non-redundant protein database with curated viral reference datasets. Corresponding reference sequences representing known viral clades and closely related taxa were retrieved from GenBank to provide phylogenetic context. Phylogenetic analyses were restricted to viral sequences with sufficient length and conserved regions to ensure reliable alignment and tree reconstruction.

Protein sequences were aligned using MAFFT v7.520 [29] with the L-INS-i algorithm, which is optimized for accuracy in datasets with variable sequence length and moderate divergence. Alignments were manually inspected and curated using AliView v1.28 [37] to verify reading frame consistency, identify poorly aligned regions, and confirm that conserved RdRp motifs were properly aligned. To reduce phylogenetic noise arising from ambiguously aligned or highly variable regions, alignments were trimmed using ClipKIT v1.3.0 [59] with the “smart-gap” strategy, which removes alignment columns with excessive gaps while retaining phylogenetically informative sites.

Maximum likelihood phylogenetic trees were reconstructed using IQ-TREE v2.2.2.6 [50]. The best-fitting amino acid substitution model for each alignment was selected automatically using ModelFinder implemented in IQ-TREE based on Bayesian Information Criterion (BIC). Branch support was assessed using 1,000 ultrafast bootstrap replicates combined with SH-like approximate likelihood ratio tests (SH-aLRT), providing robust measures of node confidence. Nodes with ultrafast bootstrap support ≥ 95% and SH-aLRT support ≥ 80% were considered strongly supported. For the *Chuviridae* dataset, the best-fit model was LG + F+R6, with a RapidNJ starting tree log-likelihood of -127,568.089 and a final optimized likelihood score of -127,382.018. For the Nairoviridae dataset, the optimal model was Q.yeast + F+R6, with a RapidNJ log-likelihood of -204,087.415 and a final likelihood of -203,064.648. For Partitiviridae, ModelFinder selected LG + I+G4, yielding a RapidNJ log-likelihood of -19,180.027 and a final likelihood of -19,112.164. For Phenuiviridae, the best-fit model was Q.yeast + F+R7, with a RapidNJ log-likelihood of -181,883.814 and a final optimized likelihood of -181,478.618.

Trees were inferred as either unrooted or rooted depending on the availability of appropriate outgroup sequences. When suitable distant homologs were available, trees were rooted using outgroup taxa belonging to related viral families; otherwise, midpoint rooting was applied. Tree topologies were visualized and annotated using Interactive Tree Of Life (iTOL) v6 [39], allowing integration of metadata such as tick species, geographic origin, and viral family assignments. Branches corresponding to Finnish sequences were highlighted to facilitate comparison with previously described viral lineages.

Phylogenetic placement of Finnish viral sequences was used to assess taxonomic relationships, identify clustering with known tick-associated virus clades, and evaluate genetic divergence relative to established species. Sequences that formed well-supported monophyletic groups distinct from described taxa were considered putative novel or divergent viral lineages, pending formal taxonomic classification following ICTV guidelines.

## Results

### Read processing and quality control

Metatranscriptomic sequencing was completed for 92 individual adult ticks, comprising 46 *Ixodes ricinus* (23 females and 23 males) and 46 *Ixodes persulcatus* (23 females and 23 males). All libraries passed platform quality-control metrics, and no samples were excluded from downstream analyses. Mean Phred quality scores exceeded 34 across all libraries, indicating high base-calling accuracy. Following adapter removal and quality trimming, reads were further processed through the NMDC metatranscriptomic workflow. Read assembly and contig length distribution are shown in Supplementary Table 1.

Reference-guided mapping to the corresponding tick genomes resulted in mean tick-aligned fractions of 89.4% ± 3.2% for *I. ricinus* and 91.1% ± 2.6% for *I. persulcatus*. Here and throughout the results, values reported as ± represent standard deviation (SD). The remaining non-tick reads averaged 3.2 × 10⁶ ± 0.4 × 10⁶ reads per *I. ricinus* sample and 1.01 × 10⁶ ± 0.2 × 10⁶ reads per *I. persulcatus* sample and were retained for downstream microbial and viral analyses. Following quality control and removal of host-derived sequences, the average microbial component among the retained non-tick reads was approximately 1.37 × 10⁶ ± 0.1 × 10⁶ reads per *I. ricinus* sample (Supplementary Figs S1 – S3).

### Overview of viral diversity across sampled *ixodes* ticks

**Fig. 1:**
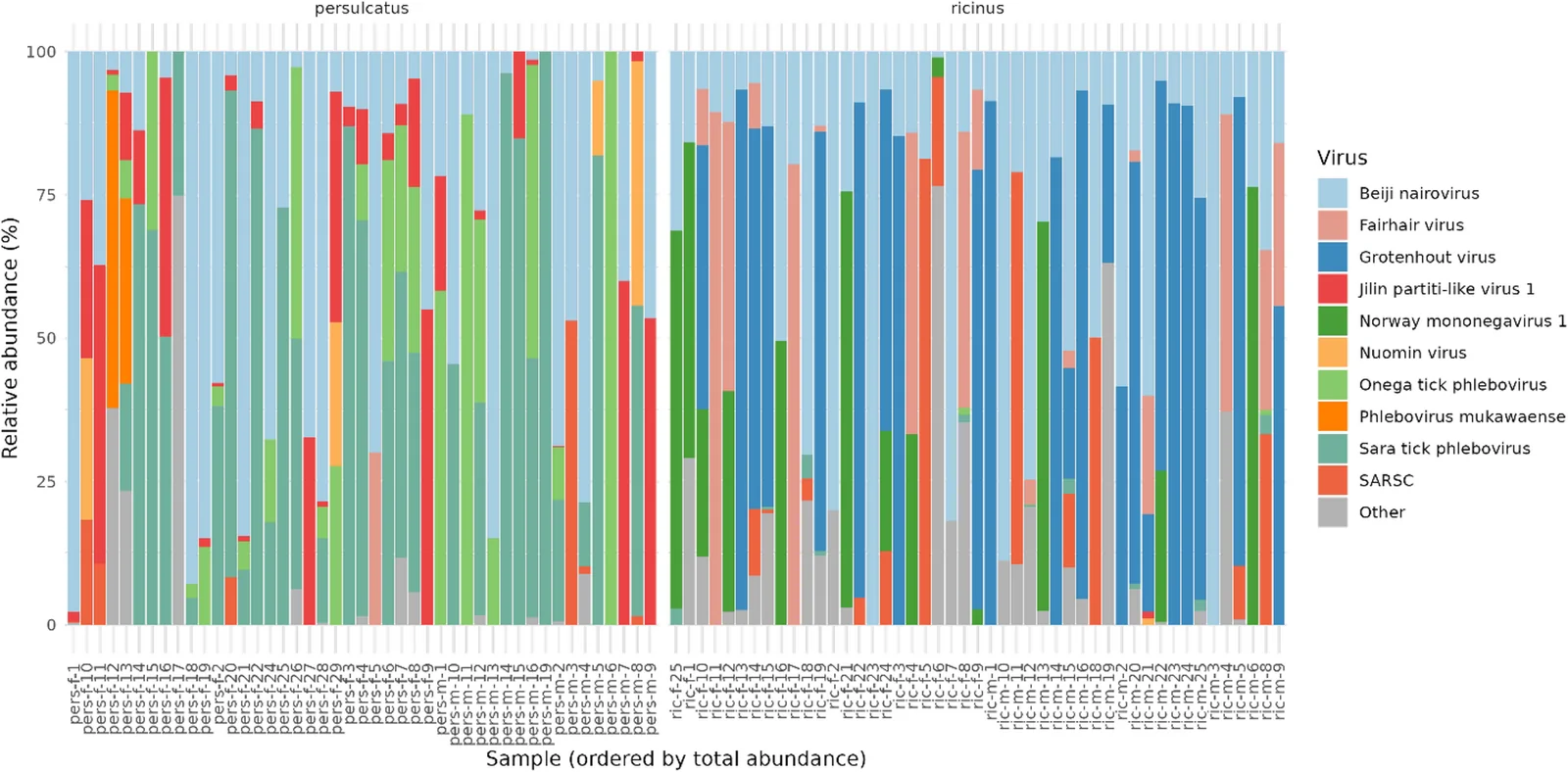
Viral community composition across all tick samples. Relative abundance (%) of the top 10 most abundant viral taxa per sample. Each bar represents an individual tick sample, ordered by total viral abundance, while colours correspond to distinct viral taxa

Across the 92 tick samples, we identified 42 distinct RNA viruses spanning 12 viral families (Supplementary Table S2). Relative-abundance profiles demonstrated substantial variation in virome composition among individual ticks, with clear differences in the representation of viral taxa between the two Ixodes species (Fig. 1).

The most represented viral families were *Nairoviridae* (11 taxa), *Phenuiviridae* (9), *Partitiviridae* (7), *Flaviviridae* (4), and *Orthomyxoviridae* (3). Individual ticks harboured an average of 10.9 ± 3.2 viral taxa, indicating considerable viral diversity at the single-tick level. Although several viruses accounted for a substantial proportion of the total viral read abundance, many other taxa occurred at lower relative abundance and prevalence, demonstrating a highly uneven distribution of viral taxa across individual ticks.

All analysed tick samples contained detectable viral sequences, although the number and relative representation of viral taxa varied considerably among individuals. The combination of high-prevalence dominant viruses and numerous less-abundant taxa indicates that the Finnish *Ixodes* virome comprises both widespread core components and rarer viral taxa.

### Prevalence and abundance structure of viral taxa

**Fig. 2:**
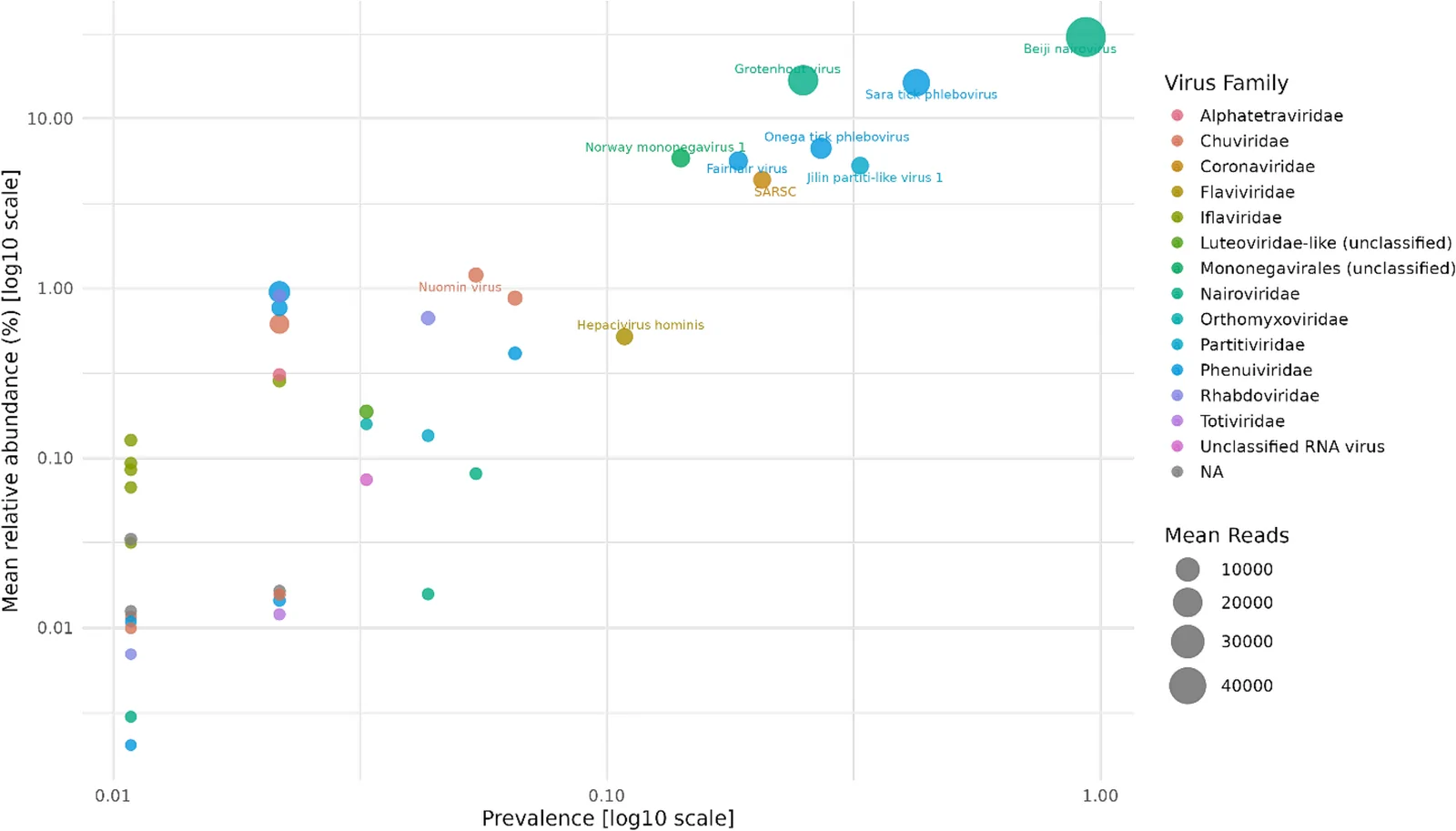
Bubble plot showing prevalence and relative abundance of the 20 most variable viral taxa. Prevalence represents the proportion of tick samples in which each virus was detected, while relative abundance represents the proportion of virus-associated reads assigned to each virus within each sample. Bubble size indicates prevalence and colour indicates viral family. Both axes are shown on a logarithmic scale

**Fig. 3:**
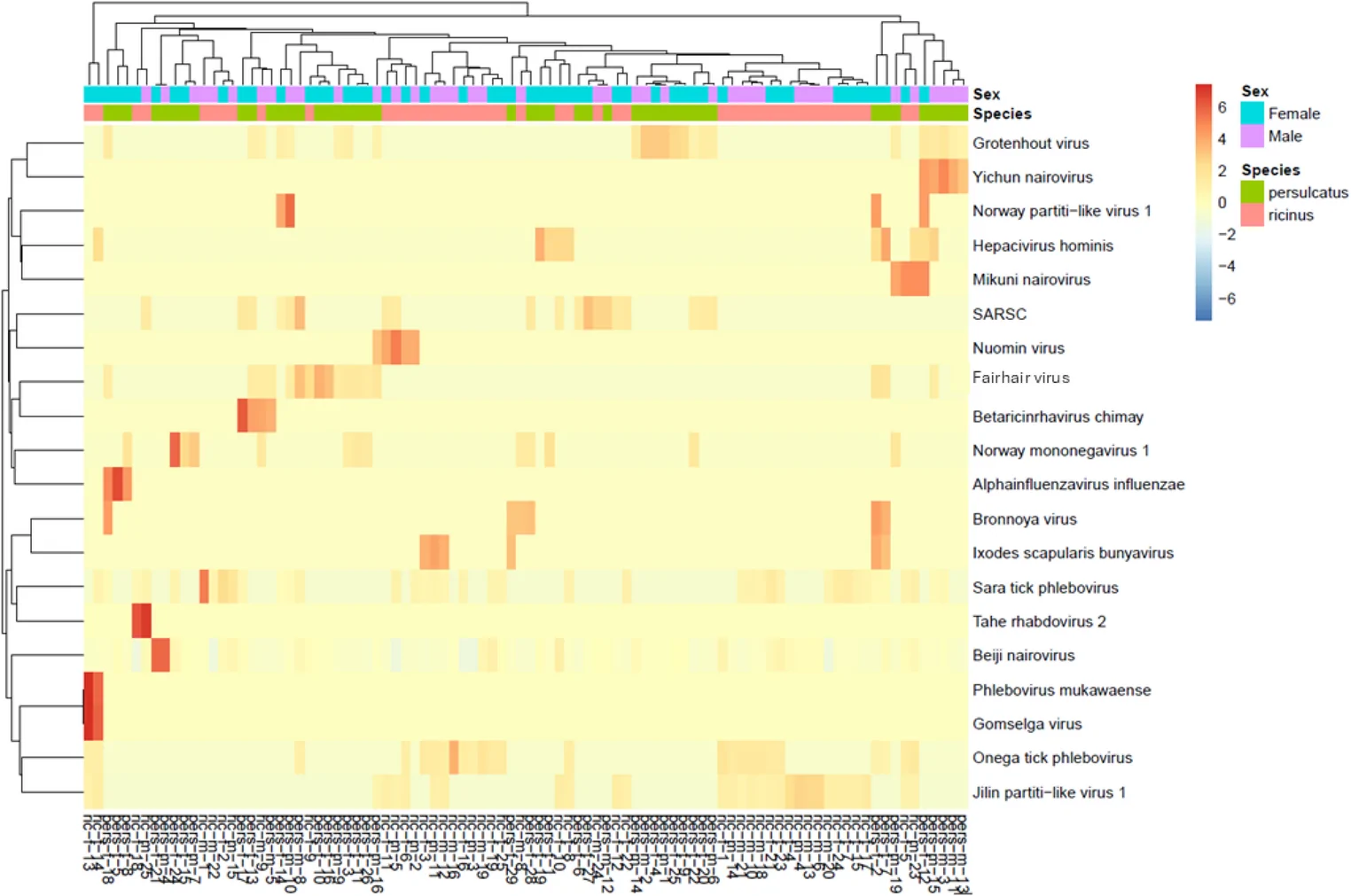
Heatmap of differentially abundant tick-associated RNA viruses between species. The heatmap displays the 20 viruses identified as significantly differentially abundant by DESeq2 analysis of normalized viruses read counts. Rows represent viruses and columns represent individual tick samples. Colour intensity corresponds to log2-transformed normalized abundance values (scaled as indicated in the legend). Differential abundance was determined using the Wald test with Benjamini-Hochberg false discovery rate (FDR) correction (adjusted p < 0.05). Patterns illustrate clear species-associated clustering of viral abundance profiles

Prevalence-abundance analysis revealed a strongly skewed distribution, with a small number of viral taxa occurring frequently across the sampled ticks, whereas many taxa were detected in relatively few individuals (Fig. 2). Nine viral taxa were detected in more than 20% of the sampled ticks. Beiji nairovirus was the most prevalent taxon, occurring in 96% of samples, followed by Grotenhout virus (89%), Sara tick phlebovirus (85%), and Onega tick phlebovirus (82%). These highly prevalent viruses also contributed substantially to the overall viral read pool. Across the complete dataset, the five taxa contributing the largest proportions of virus-associated reads were Beiji nairovirus (26.5%), Grotenhout virus (18.7%), Sara tick phlebovirus (12.4%), Onega tick phlebovirus (11.6%), and Jilin partiti-like virus 1 (15.3%) (Figs. 1, 2 and 3). These values illustrate the uneven distribution of viral reads, with a relatively small number of taxa accounting for a large fraction of the detected virome.

Low-prevalence detections included *Hepacivirus hominis* and SARS-related coronavirus-like sequences (SARSC). These signals were characterized by low read abundance and, in several cases, short or fragmentary contigs.

### Viral load, richness, alpha diversity, and community structure

**Fig. 4:**
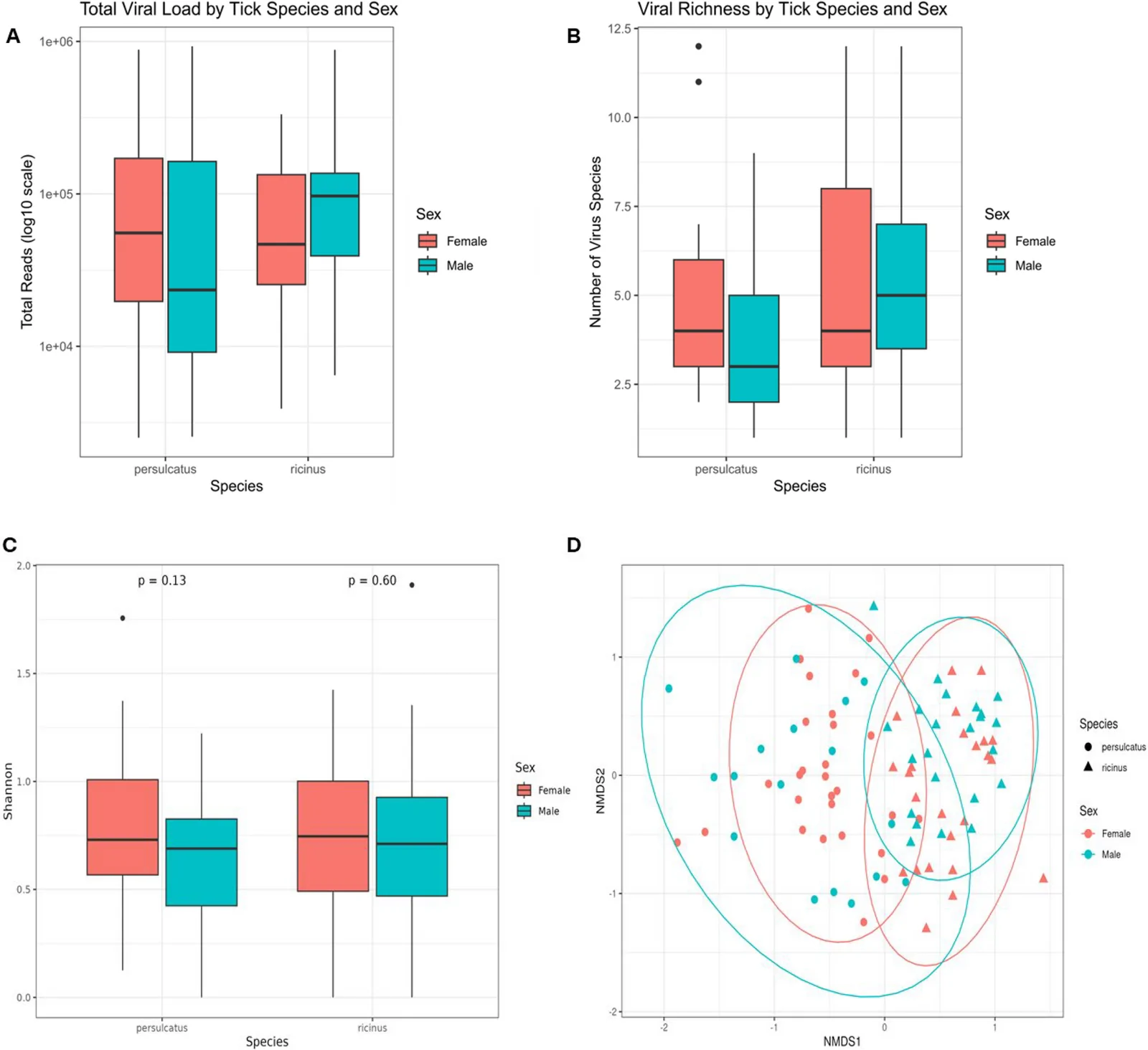
Alpha diversity and community structure of tick-associated RNA viromes. **A **& **B** total number of ticks by species based on viral read count and viral richness (number of detected viral taxa per tick). (**C**) Shannon alpha diversity of viral communities in Ixodes persulcatus and Ixodes ricinus, stratified by sex. Boxes represent interquartile ranges, centre lines indicate medians, and whiskers denote range. (**D**) Non-metric multidimensional scaling (NMDS) ordination of Bray-Curtis dissimilarities among viral communities from individual ticks (stress = 0.183). Points represent individual ticks, coloured by species and shaped by sex. Ellipses indicate 95% confidence intervals for species centroids

Total viral read abundance and viral richness varied among tick samples and between species and sex categories (Figs. 4 A & B). I. ricinus showed broader dispersion in viral abundance and richness than I. persulcatus, indicating greater inter-individual variability in virome composition. Overall, species-level differences were more pronounced than sex-associated differences (Supplementary Table S3).

Shannon diversity was significantly higher in I. ricinus (H = 2.84 ± 0.52) than in I. persulcatus (H = 2.36 ± 0.41; Welch’s t = 2.41, p = 0.013; Fig. 4C). In contrast, Simpson’s diversity did not differ significantly between species (p = 0.145). The higher Shannon diversity in I. ricinus therefore reflects differences in the combined contribution of richness and relative evenness rather than a simple difference in dominance by a single viral taxon.

Sex-associated differences were comparatively limited and varied between species. Female *I. persulcatus* ticks harboured more viral taxa on average than males (12.4 ± 2.8 versus 9.7 ± 2.2; *p* = 0.046), whereas no significant difference was detected between female and male *I. ricinus* (*p* = 0.273). Overall, sex explained less variation in virome diversity than the species-level comparison.

Community-level analysis further demonstrated significant differences in virome composition between the two tick species. PERMANOVA detected a significant effect of tick species (p = 0.001; Supplementary Table S3). Bray–Curtis dissimilarities were visualized using non-metric multidimensional scaling (NMDS), which produced an ordination with a stress value of 0.183 (Fig. 4D).

The NMDS showed partially separated viral communities between *I. ricinus* and *I. persulcatus*, with greater overlap between males and females within each species. The broader dispersion of *I. ricinus* samples was consistent with the greater inter-individual variation observed in viral richness and abundance. Together, the PERMANOVA and NMDS results support an association between tick species and overall virome composition, while the comparatively limited separation by sex indicates a weaker sex-associated effect.

### Species-specific virome profiles and differential abundance

Hierarchical clustering of viral abundance profiles revealed separation associated primarily with tick species, with less pronounced separation according to sex (Fig. 3). The observed abundance patterns showed species-associated clustering of individual tick samples.

At the viral-family level, the relative representation of major viral groups differed between the two *Ixodes* species. *I. ricinus* showed higher relative representation of *Nairoviridae* (34.8% ± 9.7%) and *Iflaviridae* (22.5% ± 7.6%), whereas *I. persulcatus* showed higher relative representation of *Phenuiviridae* (21.4% ± 5.9%) and *Partitiviridae* (18.6% ± 4.2%). The *I. persulcatus* virome was characterized by a strong contribution from Jilin partiti-like virus 1, Sara tick phlebovirus, and Onega tick phlebovirus, whereas *I. ricinus* displayed a comparatively more even distribution among several viral taxa.

Differential-abundance analysis using DESeq2 identified 15 viral taxa with significant differences between tick species after multiple-testing correction (Benjamini–Hochberg adjusted *p* < 0.05; Supplementary Table S4). Several taxa, including Jilin partiti-like virus 1, Beiji nairovirus, Onega tick phlebovirus, and Sara tick phlebovirus, showed higher relative representation in *I. persulcatus*. Conversely, Grotenhout virus, Bronnoya virus, Betaricinrhavirus chimay, and Norway partiti-like virus 1 showed higher relative representation in *I. ricinus*. These differential-abundance patterns provide evidence for species-associated differences in the.

### Phylogenetic placement of identified viral proteins

**Fig. 5:**
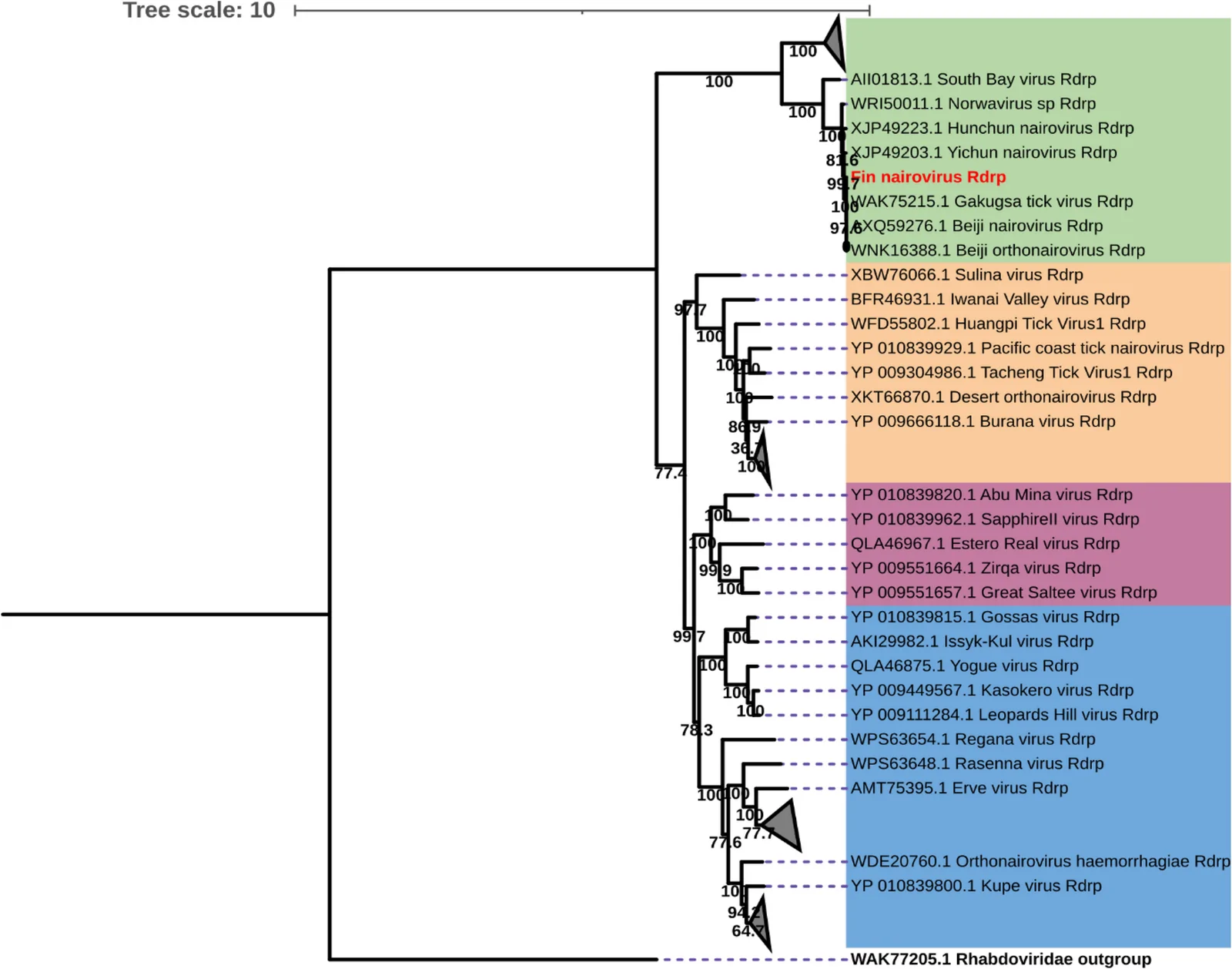
Maximum-likelihood phylogeny of *Nairoviridae* RdRp sequences. Maximum-likelihood tree inferred from RdRp amino acid sequences from NCBI RefSeq database using IQ-TREE under the best-fit model (Q.yeast + F+R6). Finnish sequences (red) cluster within tick-associated orthonairovirus clades and show high amino acid identity to Beiji orthonairovirus and Gakugsa tick virus. Branch support values are shown at key nodes. Color codes indicate viral sequence clustering based on phylogenetic analysis

**Fig. 6:**
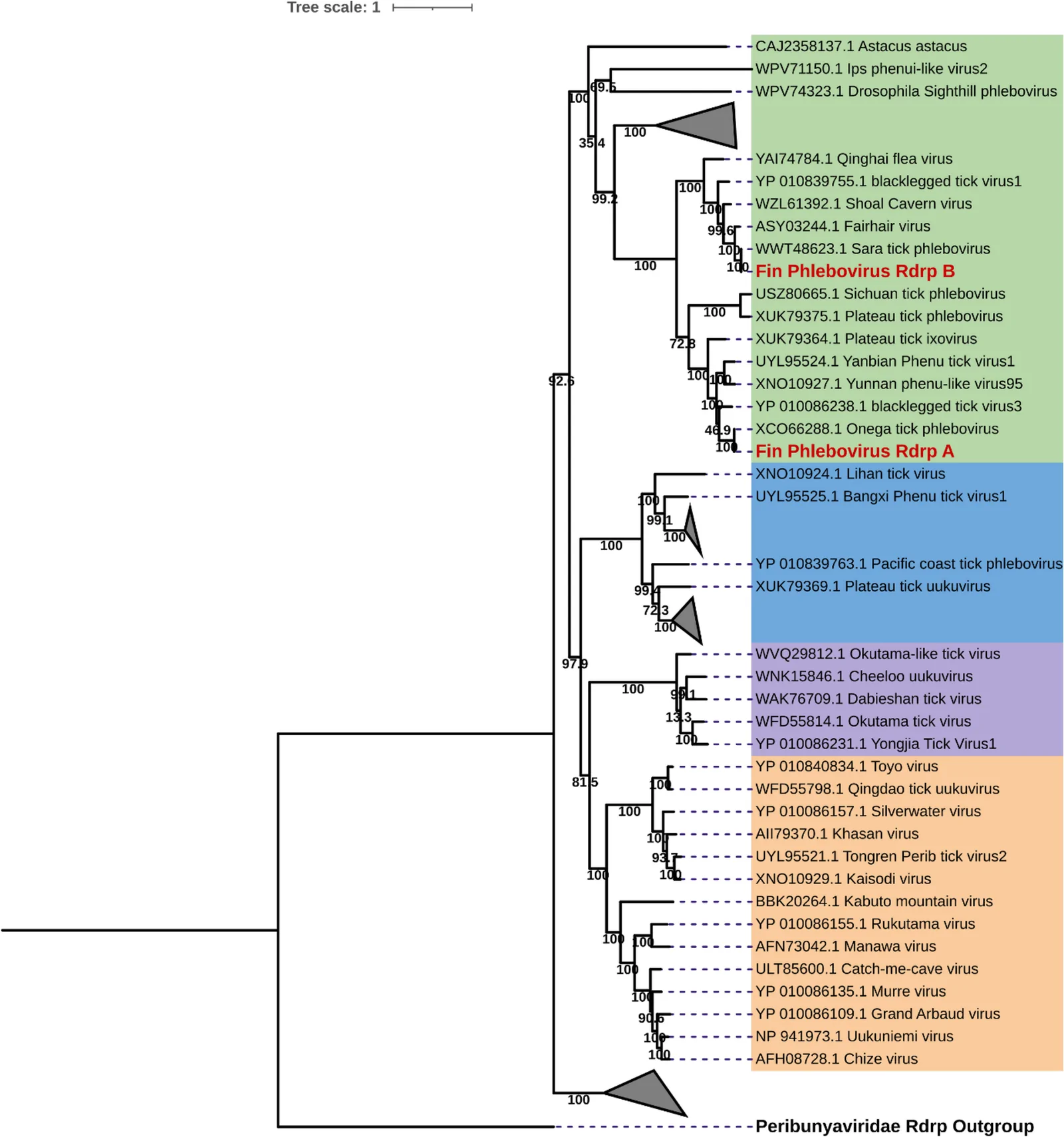
Maximum-likelihood phylogeny of Phenuiviridae RdRp sequences. Phylogenetic reconstruction of phlebovirus-related RdRp sequences from NCBI RefSeq database using IQ-TREE (model Q.yeast + F+R7). Finnish sequences (red) group with Onega tick phlebovirus and Sara tick phlebovirus lineages with high amino acid identity

**Fig. 7:**
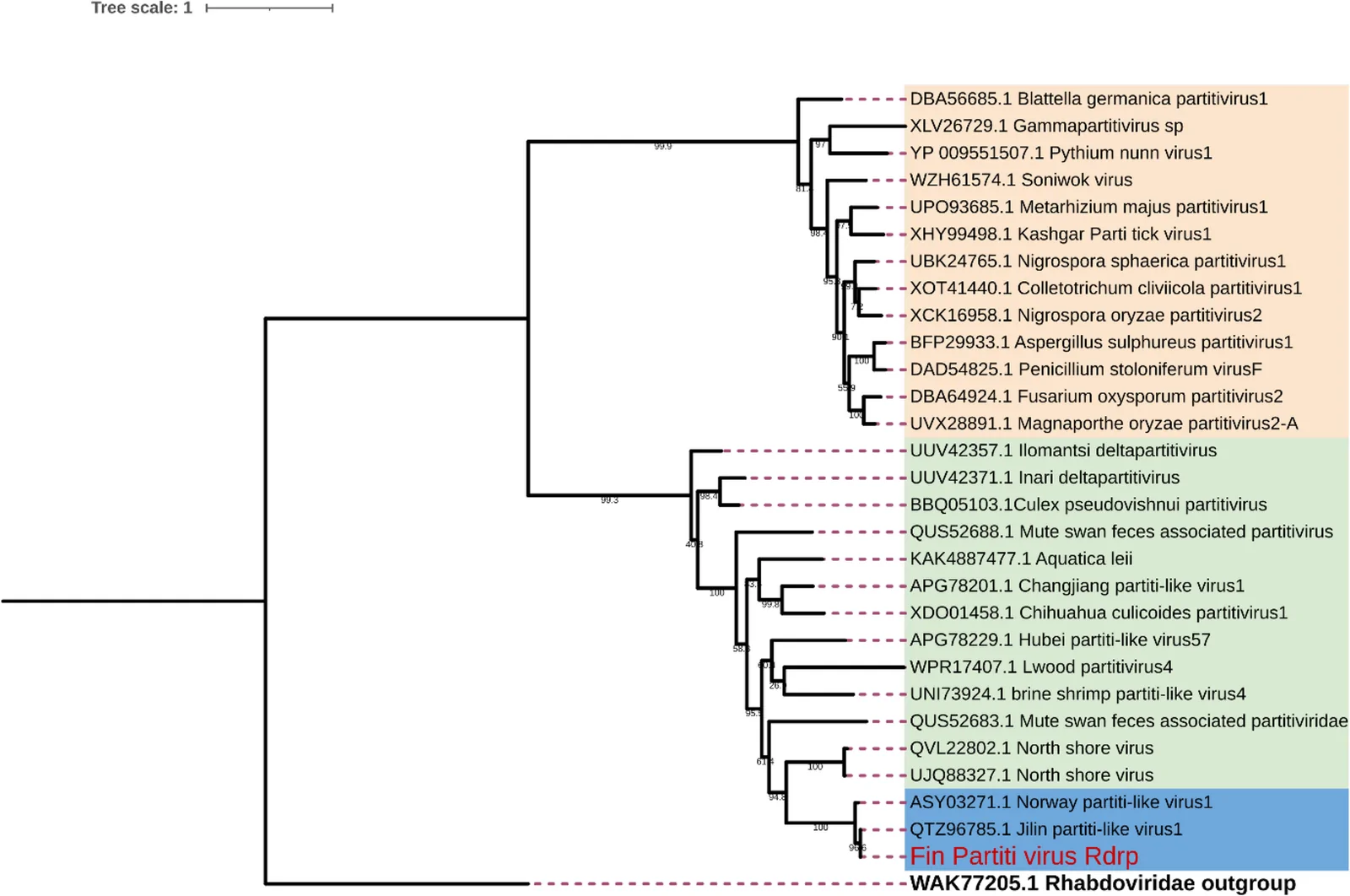
Maximum-likelihood phylogeny of *Partitiviridae*-like RdRp sequences. Maximum-likelihood tree of partiti-like RdRp sequences from NCBI RefSeq database inferred under model LG + I+G4. Finnish sequences (red) cluster with Jilin and Norway partiti-like viruses in a tick-associated clade distinct from plant and fungal partitiviruses. Branch structure supports a host-associated radiation of tick-linked partiti-like viruses

**Fig. 8:**
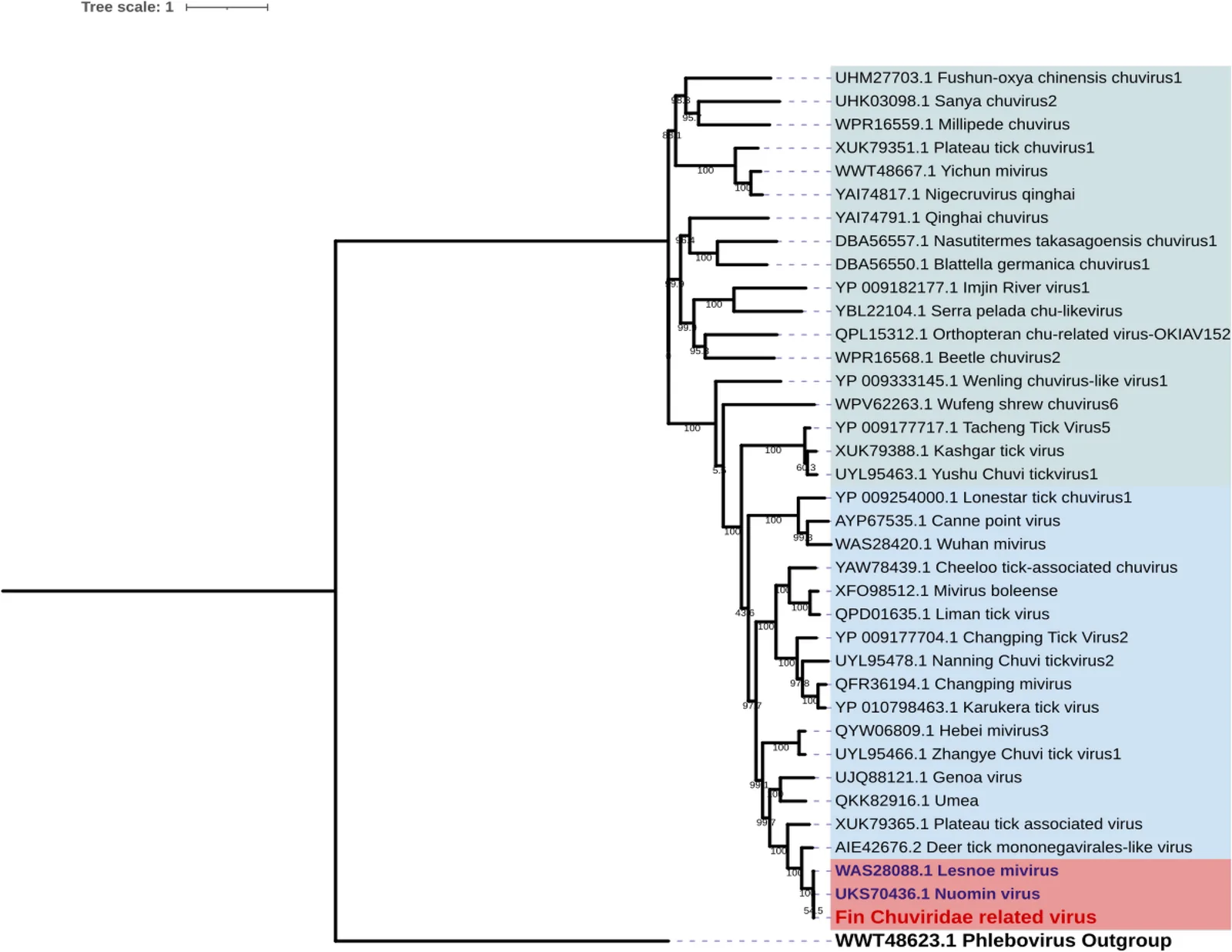
Maximum-likelihood phylogeny of *Chuviridae*-related RdRp sequences. Phylogenetic tree inferred based on RdRp sequences from the NCBI RefSeq database using the model LG + F+R6, showing placement of the Finnish chuvirus sequence within a tick-associated Chuviridae clade. The Finnish sequence clusters tightly with the Nuomin virus and Lesnoe virus (> 99% amino acid identity) and is clearly separated from more distant mononegavirales-like viruses

Maximum-likelihood phylogenetic reconstruction of RdRp amino acid sequences placed all Finnish viral sequences within established arthropod-associated viral clades (Figs. 5, 6, 7 and 8). Tree topologies were stable across model selection and bootstrap resampling, and alignments were manually inspected to remove poorly aligned regions before inference. Importantly, none of the Finnish sequences clustered within well-characterized vertebrate-adapted virus clades, supporting their classification as components of the tick-associated virome rather than misassigned vertebrate pathogens.

Finnish nairovirus sequences grouped within tick-associated orthonairovirus clades (Fig. 5). Fin_nairovirus_Rdrp showed 98.01% amino acid identity with Beiji orthonairovirus, 97.87% with Gakugsa tick virus, and 87.95% with Yichun nairovirus, supporting assignment to a Eurasian tick-associated nairovirus lineage.

Two distinct phlebovirus lineages were resolved (Fig. 6). Fin_Phlebovirus_Rdrp-A was nearly identical to Onega tick phlebovirus (99.63% identity, accession number: XCO66288.1), whereas Fin_Phlebovirus-B clustered with Sara tick phlebovirus (99.47% identity) and showed lower similarity to Fairhair virus (89.29%) and Shoal Cavern virus (78.20%). Partiti-like viruses formed a strongly supported tick-associated clade distinct from plant and fungal partitiviruses (Fig. 7). The Finnish partiti-like sequence showed 100% identity to Jilin partiti-like virus 1 and 94.42% identity to Norway partiti-like virus 1.

A Finnish chuvirus-related sequence clustered within tick-associated Chuviridae lineages (Fig. 8). The RdRp segment showed 99.36% and 99.34% identity to Nuomin virus (accession number: UKS70436.1) and Lesnoe virus (accession number: WAS28088.1), respectively, and substantially lower identity (77.24%) to Deer tick mononegavirales-like virus (accession number: AIE42676.2), supporting placement within a Nuomin-like lineage.

## Discussion

This study provides a high-resolution, single-tick metatranscriptomic comparison of the RNA viromes associated with *Ixodes ricinus* and *Ixodes persulcatus* in Finland and establishes a baseline for tick-associated RNA virus diversity in a northern European boreal environment. Across 92 individually sequenced adult ticks, we identified 42 viral RNA reads representing 12 viral families. The detection of diverse RNA viruses across both *Ixodes* species is consistent with previous metatranscriptomic surveys demonstrating that ticks harbour complex viral communities across Eurasia [52], [56], 63, [66]. The single-tick design further demonstrates that much of this diversity can be obscured when samples are pooled, highlighting the value of individual-level metatranscriptomic profiling for resolving variation in tick viromes.

### Species-associated structuring of the tick virome

A major finding of this study was the clear association between tick species and virome composition. Community-level analyses demonstrated significant differences between *I. ricinus* and *I. persulcatus*, with NMDS ordination showing partial separation of the two species (Fig. 4D). Similar species-associated structuring of tick-associated viral communities has been reported previously, suggesting that host species identity can contribute substantially to the assembly of tick viromes [46], [52]. In contrast, males and females showed substantial overlap within species, suggesting that sex had a comparatively weaker influence on overall virome structure.

The species-associated separation was accompanied by differences in alpha diversity. *I. ricinus* exhibited significantly higher Shannon diversity than *I. persulcatus*, whereas Simpson diversity did not differ significantly between species. This combination suggests that the difference in Shannon diversity was associated primarily with differences in richness and relative evenness rather than a simple difference in dominance by one viral taxon. The greater dispersion of *I. ricinus* samples in the ordination was also consistent with greater inter-individual variation in viral richness and abundance. By contrast, *I. persulcatus* samples formed a more compact cluster, suggesting comparatively similar virome profiles among individuals. Comparable differences in the structure and variability of tick-associated viral communities have been reported among Eurasian *Ixodes* populations [31], [65].

Nevertheless, the present findings should be interpreted cautiously because tick species and sampling location were not independently replicated. *I. ricinus* and *I. persulcatus* were sampled from different geographic locations; consequently, species and location effects are confounded. The observed differences therefore demonstrate an association between species and virome composition but cannot establish that tick species identity alone caused the observed community differentiation. Sampling both species sympatrically across multiple locations would provide a stronger test of species-specific virome structuring.

### Differences in viral community composition

The taxonomic composition of the viromes further supported differences between the two *Ixodes* species. *I. ricinus* showed greater relative representation of *Nairoviridae* and *Iflaviridae*, whereas *I. persulcatus* showed greater representation of *Phenuiviridae* and *Partitiviridae*. These differences were accompanied by substantial variation in the abundance of individual viral taxa (Figs. 1, 2 and 3). The analyses show that a relatively small number of highly abundant taxa contributed disproportionately to the total viral read pool, whereas other taxa were characterized by lower prevalence and greater variability among individual ticks (Fig. 2).

Beiji nairovirus, Grotenhout virus, Sara tick phlebovirus, and Onega tick phlebovirus were among the most prevalent viruses in the dataset, while Jilin partiti-like virus 1 also contributed substantially to overall viral read abundance. The high prevalence of several of these taxa suggests that they may represent relatively common components of the RNA viromes of Finnish *Ixodes* ticks. Similar tick-associated nairovirus and phlebovirus lineages have been reported from Eurasian *Ixodes* populations, supporting their broad geographic occurrence [31], [33], [40], [60]. However, high prevalence should not be interpreted as evidence of active viral replication or vector competence, because detection of viral RNA alone does not demonstrate productive infection or transmission.

### Phylogenetic relationships of finnish tick-associated viruses

Phylogenetic analyses provided additional context for selected viral groups detected in Finnish ticks. Rather than constructing phylogenies for every detected taxon, analyses were performed for viral groups for which sufficiently informative RdRp sequences and appropriate reference sequences were available. The resulting trees included *Nairoviridae*, Phenuiviridae, *Partitiviridae*-like viruses, and *Chuviridae*-related sequences (Figs. 5, 6, 7 and 8).

Finnish nairovirus sequences clustered within tick-associated orthonairovirus lineages and showed high amino acid similarity to previously described Eurasian tick-associated viruses. In particular, the Finnish nairovirus sequence showed 98.01% amino acid identity to Beiji orthonairovirus and 97.87% to Gakugsa tick virus, with lower similarity to Yichun nairovirus. These relationships support the placement of the Finnish sequences within established tick-associated nairovirus diversity (Fig. 5).

The close relationship between the Finnish nairovirus sequences and previously reported Beiji/Gakugsa-like viruses is consistent with recent evidence that related Norwavirus lineages occur across geographically separated *Ixodes* populations in Eurasia [31], [65]. Importantly, current ICTV taxonomy recognizes *Norwavirus beijiense* and *Norwavirus grotenhoutense* as distinct species, while several historically used virus names are treated as synonyms or discouraged alternative designations. Taxonomic curation is therefore essential when interpreting the apparent number of viral taxa and their geographic distributions.

Similarly, two Finnish phlebovirus-related sequences were closely associated with previously described tick-associated lineages. One sequence showed 99.63% amino acid identity to Onega tick phlebovirus, whereas the second clustered with Sara tick phlebovirus and showed 99.47% amino acid identity. These relationships are consistent with previous reports of tick-associated phleboviruses from Eurasian *Ixodes* populations [31], [33].

The Finnish partiti-like sequences also clustered with previously reported tick-associated partiti-like viruses, showing 100% amino acid identity to Jilin partiti-like virus 1 and 94.42% identity to Norway partiti-like virus 1 (Fig. 7). This phylogenetic placement is consistent with the growing recognition of arthropod-associated partiti-like viruses as a component of tick viromes [8], [56].

The Finnish chuvirus-related sequence showed similarly close relationships to Nuomin virus and Lesnoe virus, with 99.36% and 99.34% amino acid identity, respectively. Closely related chuvirus lineages have previously been detected in ticks and other arthropod-associated samples, demonstrating the broad diversity of negative-sense RNA viruses associated with arthropods [56].

Overall, these phylogenetic relationships indicate that several Finnish viral sequences are closely related to previously described Eurasian tick-associated viruses. The results therefore extend the known geographic occurrence of these lineages rather than providing sufficient evidence for the designation of new viral species. Where sequences remain unresolved because of limited sequence length, database representation, or insufficient phylogenetic resolution, they are more appropriately described as unclassified or putatively divergent lineages rather than formally novel viruses.

### Geographic distribution and ecological interpretation

The detection in Finland of viral lineages previously reported elsewhere in Eurasia extends the known geographic occurrence of these viruses and demonstrates the value of northern European tick populations for understanding the distribution of tick-associated RNA viruses. Similar geographic patterns have been documented for tick-associated viruses across Europe and Asia, where closely related viral lineages occur across broad geographic ranges [31], [], [], [55].

The presence of related viral lineages across geographically separated tick populations could reflect long-standing associations between viruses and their tick hosts, broad distributions of competent vertebrate hosts, historical dispersal, or contemporary movement of vectors and hosts [55, 56]. However, the present study cannot distinguish among these mechanisms. In particular, migratory birds should not be invoked as the primary explanation for the geographic distribution of the detected viruses without independent evidence of bird-associated dispersal. Similarly, climate-driven range expansion may influence the distribution of *Ixodes* ticks and their associated viruses, but this study does not directly test the contribution of climate or recent range expansion to viral dispersal.

Ecological differences between *I. ricinus* and *I. persulcatus* may nevertheless contribute to the observed patterns. The two species differ in geographic distribution, habitat associations, seasonal ecology, and host interactions, factors that could influence opportunities for viral acquisition and maintenance [10], [20], [66]. The comparatively compact virome of *I. persulcatus* may reflect more consistent ecological exposure, whereas the greater dispersion observed among *I. ricinus* samples may be consistent with more heterogeneous viral acquisition. Similar associations between tick ecology and virome composition have been reported in other Eurasian tick species [27], [67].

### Implications for viruses of potential zoonotic relevance

Several detected viruses warrant continued surveillance, but their potential public health relevance should be interpreted conservatively. Beiji nairovirus and Yichun nairovirus occurred within tick-associated nairovirus diversity, while the broader *Nairoviridae* includes viruses capable of infecting vertebrates. Some nairoviruses are associated with human disease, demonstrating that this viral family contains lineages of established zoonotic importance [44, 68]. However, the detection of a tick-associated nairovirus in Finland does not itself demonstrate human pathogenicity, transmission, or vector competence.

Similarly, although *Nairoviridae* and *Phenuiviridae* include important human pathogens such as Crimean-Congo haemorrhagic fever virus and severe fever with thrombocytopenia syndrome virus, family-level relatedness alone does not imply equivalent pathogenic or zoonotic potential [57]. The Finnish sequences analysed here clustered within distinct tick-associated lineages rather than with recognized high-consequence vertebrate pathogens. Consequently, their family-level relationship to pathogenic viruses should not be interpreted as evidence of a direct public health risk.

The close phylogenetic relationship of the Finnish chuvirus-related sequence to Nuomin viruses is similarly of evolutionary and surveillance interest rather than evidence of pathogenicity. Further studies involving complete genome characterization, prevalence surveys in additional tick populations, experimental infection studies, and, where appropriate, serological investigations would be necessary to assess biological significance.

### Low-abundance vertebrate-associated viral signals

Low-abundance reads assigned to vertebrate-associated or clinically familiar viruses require particular caution. *Hepacivirus hominis*-like sequences and sporadic SARS-related coronavirus-like and *Alphainfluenzavirus influenzae* signals were detected at low abundance and lacked the genome-wide coverage required to support confident interpretation as active tick infections. Such signals could arise from environmental contamination, laboratory or reagent-associated contamination, index hopping or carryover, or vertebrate-derived material associated with a recent blood meal.

Because the present study did not experimentally demonstrate replication or establish the origin of these sequences, we do not interpret them as evidence that *Ixodes* ticks harbour or transmit these viruses. Their detection instead illustrates the sensitivity of metatranscriptomic sequencing and the importance of stringent validation of low-abundance signals [63, 64]. Similar incidental detection of vertebrate-associated viral sequences has been reported in arthropod metatranscriptomic surveys, where environmental material, host-derived material, and technical contamination can complicate interpretation [49], [56]. Future studies incorporating extraction blanks, library controls, technical replicates, host identification, and targeted molecular confirmation would be valuable for distinguishing genuine biological signals from contamination or incidental vertebrate-derived RNA.

## Conclusion

Overall, our results demonstrate that individual *I. ricinus* and *I. persulcatus* ticks in Finland harbour diverse and compositionally structured RNA viromes. The differences in community composition, together with differences in alpha diversity and the relative representation of major viral families, indicate that the two *Ixodes* species are associated with distinct viral assemblages. However, the following limitation should be considered when interpreting findings: the two tick species were collected from different geographic locations, making species and location effects inseparable in the current design. The observed species-associated differences in virome composition therefore cannot be interpreted as exclusively species-driven. Despite the limitations, the study provides an important baseline for RNA virus diversity in Finnish *Ixodes* ticks. Establishing this baseline is particularly relevant in northern Europe, where changes in tick distributions, host communities, and environmental conditions may alter opportunities for viral maintenance and transmission. Repeated sampling across Finland, including sympatric populations of *I. ricinus* and *I. persulcatus*, would allow future studies to separate species, geographic, seasonal, and environmental effects and determine whether the virome patterns observed here are stable through time.

## Supplementary Information

Below is the link to the electronic supplementary material.

Supplementary Material 1 (DOCX 418 KB)

## Data Availability

Raw sequencing data have been deposited in the NCBI Sequence Read Archive under the BioProject accession number PRJNA1444502: [https://www.ncbi.nlm.nih.gov/sra/PRJNA1444502](https:/www.ncbi.nlm.nih.gov/sra/PRJNA1444502) . Analysis scripts and workflow documentation are available at GitHub repository: [https://github.com/theoalal/Bioinformatics\_23.git](https:/github.com/theoalal/Bioinformatics_23.git) .
