# Supplementary material for: Meta-transcriptomic profiling of Ixodes ricinus and Ixodes persulcatus ticks from Finland reveals diverse RNA viromes, community structuring, and emerging viral lineages
Source: Arch Virol. 2026 Sep 21;171(10):281. doi: 10.1007/s00705-026-06741-y (PMC13593680; doi:10.1007/s00705-026-06741-y)
Supplement: Supplementary file 1 [file 705_2026_6741_MOESM1_ESM.docx]

**Supplementary for:**

**Meta-transcriptomic profiling of *Ixodes ricinus and Ixodes persulcatus* ticks reveals diverse RNA viromes, community structuring, and emerging viral lineages**

Theophilus Yaw Alale^1^, Jesse Mänttäri^1^, Heidi Viitaniemi^1^, Mikhail Ozerov^1,2^, Miguel Baltazar-Soares^1^, Jani J. Sormunen^1,3^, Eero J. Vesterinen^1,2^

1) Department of Biology, University of Turku, Turku, Finland.
2) Bioname Ltd, Itäinen Pitkäkatu 2, 20520, Turku, Finland
3) School of Medical, Molecular and Forensic Sciences, Murdoch University, Perth, Western Australia, Australia

This supplement contains the following figures and tables:

Contents

[Figure S1. Tick read removal efficiency.	2](#_Toc225700183)

[Figure S2. Tick read removal efficiency.	2](#_Toc225700184)

[Figure S3. Non-tick read QC.	3](#_Toc225700185)

[Figure. S4. Mean relative abundance	4](#_Toc225700186)

[Figure S5. Heatmap of viral taxa across individual tick samples.	4](#_Toc225700187)

[Table S1. Viruses and their family names.	5](#_Toc225700188)

[Table S2: Assembly and contig length distribution.	7](#_Toc225700189)

[Table S3. PERMANOVA (adonis) to test if communities differ by species/sex.	7](#_Toc225700190)

[Table S4. Differential abundance analysis of tick-associated RNA viruses between Ixodes ricinus and Ixodes persulcatus.	8](#_Toc225700191)

## Fig S1. Tick read removal efficiency.

Proportion of mapped and unmapped reads in *Ixodes persulcatus* samples. Bar plots illustrating the percentage of reads mapped to the *I. persulcatus* reference genome and unmapped reads across individual tick samples. Unmapped reads were retained for subsequent viral discovery and taxonomic classification.

## Fig S2. Tick read removal efficiency.

Proportion of mapped and unmapped reads in *Ixodes ricinus* samples. Bar plots showing the percentage of sequencing reads mapped to the *I. ricinus* reference genome (host reads) and unmapped reads retained for downstream metatranscriptomic analysis across individual tick samples. Unmapped reads represent non-host sequences, including viral, microbial, and residual environmental RNA.

## Fig S3. Non-tick read QC.

Summary composition of non-tick reads after fastqc. Blue bars indicate input and output reads, while red bars indicate composition of reads after tick read filtering.

## Table S1: Assembly and contig length distribution.

Length distribution of viral contigs retained after assembly and filtering thresholds.

## Table S2. Viruses and their family names.

Unclassified groups were assigned to hypothetical families

| Virus Name | Assigned Virus Family | |
| --- | --- | --- |
| Severe acute respiratory syndrome-related coronavirus | *Coronaviridae* | |
| Beiji nairovirus | *Nairoviridae* | |
| Jilin partiti-like virus 1 | *Partitiviridae* (unclassified) | |
| Hepacivirus hominis | *Flaviviridae* | |
| Kuriyama virus | *Phenuiviridae* (Phlebovirus-related) | |
| Onega tick phlebovirus | *Phenuiviridae* (Phlebovirus) | |
| Gomselga virus | *Nairoviridae* | |
| Sichuan tick toti-like virus | *Totiviridae* (unclassified) | |
| Alxa tick phlebovirus | *Phenuiviridae* (Phlebovirus) | |
| Phlebovirus dashliense | *Phenuiviridae* (Phlebovirus) | |
| Phlebovirus mukawaense | *Phenuiviridae* (Phlebovirus) | |
| Ixodes scapularis associated virus 1 | *Chuviridae* (unclassified) | |
| Sara tick phlebovirus | *Phenuiviridae* (Phlebovirus) | |
| Tahe rhabdovirus 2 | *Rhabdoviridae* | |
| Ixodes scapularis bunyavirus | *Phenuiviridae* (Bunyavirales unclassified) | |
| Mikuni nairovirus | *Nairoviridae* | |
| Fairhair virus | *Phenuiviridae* | |
| Ixodes scapularis iflavirus | *Iflaviridae* | |
| Pedaselga virus | *Chuviridae* (unclassified) |  |
| Wuhu Rhabd tick virus 1 | *Rhabdoviridae* | |
| Nuomin virus | *Chuviridae* (Mivirus-related) | |
| Norway mononegavirus 1 | Mononegavirales (unclassified) | |
| Bronnoya virus | *Nairoviridae* | |
| Betaricinrhavirus chimay | *Rhabdoviridae* | |
| Grotenhout virus | *Nairoviridae* | |
| Norway luteo-like virus 3 | *Luteoviridae* (unclassified) | |
| Norway partiti-like virus 1 | *Partitiviridae* (unclassified) | |
| Yichun nairovirus | *Nairoviridae* | |
| Sopafab virus | *Orthornavirae* | |
| Alphainfluenzavirus influenzae | *Orthomyxoviridae* | |

**Table S3. PERMANOVA (adonis) to test whether communities differ by species/sex.**

This uses Bray-Curtis dissimilarity. PERMANOVA: Permutation test for adonis under reduced model.

**(A)**

|  | Df | SumOfSqs | R2 | F | Pr(>F) |  |
| --- | --- | --- | --- | --- | --- | --- |
| Model | 3 | 5.476327 | 0.158677 | 5.532369 | 0.001 |  |
| Residual | 88 | 29.03619 | 0.841323 |  |  |  |
| Total | 91 | 34.51252 | 1 |  |  |  |

adonis2(formula = mat_numeric ~ Species*Sex, data = meta, permutations = 999, method = "bray”

(B)

|  | Df | SumOfSqs | R2 | F | Pr(>F) |
| --- | --- | --- | --- | --- | --- |
| Model | 1 | 0.333 | 0.00964 | 0.8761 | 0.564 |
| Residual | 90 | 34.180 | 0.99036 |  |  |
| Total | 91 | 34.513 | 1.00000 |  |  |

adonis2(formula = mat_numeric ~ Sex, data = meta, permutations = 999, method = "bray")

(C)

|  | Df | SumOfSqs | R2 | F | Pr(>F) |
| --- | --- | --- | --- | --- | --- |
| Model | 1 | 4.909 | 0.14225 | 14.926 | 0.001 *** |
| Residual | 90 | 29.603 | 0.85775 |  |  |
| Total | 91 | 34.513 | 1.00000 |  |  |

adonis2(formula = mat_numeric ~ Species, data = meta, permutations = 999, method = "bray"). Signif. codes: 0 ‘***’ 0.001 ‘**’ 0.01 ‘*’ 0.05 ‘.’ 0.1 ‘ ’ 1

## Table S4. Differential abundance analysis of tick-associated RNA viruses between Ixodes ricinus and Ixodes persulcatus.

Results were generated using DESeq2 based on normalized virus read counts. The table includes log2 fold change (log2FC), standard error (lfcSE), Wald test statistic (stat), raw p-values, and Benjamini-Hochberg false discovery rate adjusted p-values (padj). Positive log2FC values indicate higher abundance in *I. ricinus*, whereas negative values indicate higher abundance in *I. persulcatus*. Viruses with adjusted p< 0.05 were considered significantly differentially abundant.

|  | baseMean | log2FoldChange | lfcSE | stat | pvalue | padj |
| --- | --- | --- | --- | --- | --- | --- |
| Norway mononegavirus 1 | 829.7417 | -30 | 2.624047 | -11.4327 | 2.87E-30 | 4.88E-29 |
| Nuomin virus | 87.52466 | 28.88667 | 2.90237 | 9.952785 | 2.45E-23 | 2.08E-22 |
| Hepacivirus hominis | 28.01185 | -27.2862 | 2.90236 | -9.40139 | 5.38E-21 | 3.05E-20 |
| Betaricinrhavirus chimay | 17.45511 | -26.6546 | 2.902552 | -9.18317 | 4.19E-20 | 1.78E-19 |
| Bronnoya virus | 14.52959 | -26.3913 | 2.902655 | -9.09211 | 9.71E-20 | 3.30E-19 |
| Alphainfluenzavirus influenzae | 10.79061 | -25.9982 | 2.902867 | -8.95605 | 3.37E-19 | 9.53E-19 |
| Norway partiti-like virus 1 | 9.540346 | -25.8263 | 2.902974 | -8.89649 | 5.76E-19 | 1.40E-18 |
| Yichun nairovirus | 8.286848 | -25.6395 | 2.903114 | -8.83173 | 1.03E-18 | 2.19E-18 |
| Norway luteo-like virus 3 | 5.672775 | -25.1254 | 2.903601 | -8.6532 | 5.01E-18 | 9.46E-18 |
| Sopafab virus | 2.415553 | -23.9312 | 2.905497 | -8.23652 | 1.77E-16 | 3.01E-16 |
| Jilin partiti-like virus 1 | 4350.588 | 10.55723 | 1.384944 | 7.622857 | 2.48E-14 | 3.83E-14 |
| Grotenhout virus | 17195.38 | -12.052 | 1.600286 | -7.53118 | 5.03E-14 | 7.12E-14 |
| Onega tick phlebovirus | 4690.911 | 8.889789 | 1.626752 | 5.464746 | 4.64E-08 | 6.06E-08 |
| Sara tick phlebovirus | 20203.61 | 5.576302 | 1.269066 | 4.394022 | 1.11E-05 | 1.35E-05 |
| Fairhair virus | 450.5803 | -5.57591 | 2.431984 | -2.29274 | 0.021863 | 0.024778 |
| Beiji nairovirus | 73929.98 | -0.83919 | 0.594519 | -1.41154 | 0.158086 | 0.167966 |
| SARSC | 365.9961 | -1.04711 | 2.304778 | -0.45432 | 0.649599 | 0.649599 |
